# An efficient approach for the synthesis of novel methyl sulfones in acetic acid medium and evaluation of antimicrobial activity

**DOI:** 10.3906/kim-2003-10

**Published:** 2020-10-26

**Authors:** Gollapudi RAVI KUMAR, Chandra Rao DASIREDDY, Ravi VARALA, Vijay KOTRA, Hari Babu BOLLIKOLLA

**Affiliations:** 1 Department of Chemistry, Acharya Nagarjuna University, Guntur, Andhra Pradesh India; 2 Department of Chemistry, Government Degree College, Husnabad, Telangana India; 3 CSIR-Indian Institute of Chemical Technology (IICT), Hydearabad, Telangana India; 4 Retention Chromatography & Chemicals Pvt. Ltd., Secunderabad, Telangana India; 5 Scrips Pharma, Hyderabad, Telangana India; 6 Department of Pharmaceutical Chemistry, Faculty of Pharmacy, Quest International University Perak, Ipoh, Negeri Perak Malaysia

**Keywords:** BF _3_ .OEt _2_, allyl alcohols, methyl sulphones, antibacterial, antifungal

## Abstract

A series of nine methyl sulphones ( **3a** –3 **i** ) starting from the aldehydes ( **1a–1i** ) were synthesized in two consecutive steps. In the first step, preparation of allyl alcohols ( **2a–2i** ) from their corresponding aldehydes by the reaction of sodium borohydride in methanol at room temperature is reported. Finally, methyl sulphones are synthesized by condensing sodium methyl sulfinates with allyl alcohols in the presence of BF _3_ .Et _2_ O in acetic acid medium at room temperature for about 2–3 h. The reaction conditions are simple, yields are high (85%–95%), and the products were obtained with good purity. All the synthesized compounds were characterized by their ^1^ H, ^13^ C NMR, and mass spectral analysis. All the title compounds were screened for antimicrobial activity. Among the compounds tested, the compound **3f** has inhibited both Gram positive and Gram negative bacteria effectively and compound **3i** has shown potent antifungal activity. These promising components may help to develop more potent drugs in the near future for the treatment of bacterial and fungal infections.

## 1. Introduction

The alcohol functional group is one of the more important groups for the synthesis of many drugs which are being used widely throughout the world [1–5]. As the alcohol functional group is not a good leaving group, it becomes the main obstacle for producing versatile novel drugs in organic synthesis. The nucleophilic substitution in the alcohol group is very difficult under mild conditions [6–12]. For the replacement of the OH group, one has to convert this alcohol group into a Cl group which is a better leaving group. In the previous studies, it was revealed that the conversion of the OH group into a mesylate group took place [13]. Direct conversion of alcohols into ethers, diaryl alkanes, and sulphonamides was successful [14–16]. So, we have decided to optimize the convenient route for the conversion of alcohols into sulfones under mild conditions. Earlier works revealed that direct conversion of alcohols into sulfones using bronsted acids like formic acids, acetic acid, and HCl [17–20], could be generated from sodium sulfide, sodium sulfinates, sulfonic acids, potassium meta bisulfite, sulfonyl chloride, and arenesulfonyl cyanide [21–27]. Among these reagents, sodium sulfinate is the best reagent due to ease of handling, and from a stability point of view.

Reddy and co-workers [28–29] reported that the reaction between p-toluenesulfonyl cyanide, and allylic alcohols leads to the formation of p-toluenesulfonyl cyanide, in the presence of diisopropylethylamine, later the adduct gets converted into a sulfonyl rearrangement product. Direct substitution of the allylic amine with sodium sulfinates in the presence of boronic acid [30] and the use of FeCl _3_ as a catalyst and chlorotrimethylsilane as an additive [31], were also reported. Direct substitution of alcohols in the presence of boron trifluoride etherate with sodium sulfinates was prepared in which dichloromethane as a solvent was used under optimized parameters at 50 °C with 82% yield [32]. Oxidation of the methylthio derivative to the corresponding sulfones using m-CPBA was reported by Pujol et al [33]. Cu-catalyzed aerobic oxidation to synthesize from aryl halides and DMSO is described by Yuan et al. [34]. Fe(OH) _3_ -catalyzed synthesis of aryl sulfones using aryl sulfonyl chloride with arenes is also reported [35]. The L-Proline sodium salt/CuI-mediated coupling reaction of aryl halides with sulfinic acids is also documented by Ma and Zhu [36]. Yuan et al. have recently synthesized aryl ethyl sulfones from sodium sulfinate and di- *tert-* butyl peroxide in the H _2_ O medium [37]. Very recently, an eco-friendly approach to the construction of aryl methyl sulfone from SO _2_ and methyl reagents is exemplified by Jiang et al. [38]. An excellent review on sulfones was presented very recently by Trost et al. [39]. In view of this and as an extension to our search for novel antimicrobial agents [40–44], the authors herein made an attempt to synthesize the titled sulfones and screen their antimicrobial properties.

## 2. Present work

**Scheme 1:**
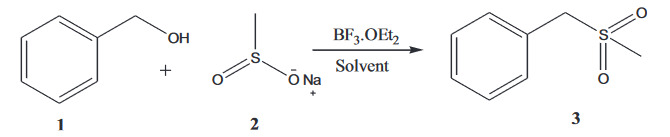
Synthesis of aryl methylsulfones.

We synthesized allyl alcohols, which were derived from respective aldehydes by reduction with sodium borohydride. A total of 9 aryl methyl sulfones were synthesized using BF _3_ .OEt _2_ as a catalyst and AcOH as a solvent in the present methodology shown in Scheme 1.

Huang et al. [32] reported on the synthesis of sulfinates using BF _3_ .OEt _2_ in CH _2_ Cl _2_ solvent medium optimized at 45–50 °C moderate temperatures via the more favourable SN ^1^ mechanism through the conversion of sodium p-toluenesulfinate into corresponding nucleophile sulfinic acid, i.e. O-attack. Surprisingly, when acetic acid was used as a solvent, we could observe the formation of sulfones possibly via S-attack following the SN ^2^ mechanism, thereby indicating the significant role of solvent in product formation. The aim was achieved with various benzyl alcohols and sodium methyl sulfinates. Shorter reaction times and direct isolation of products were the added advantages in using the previous method.

Baidya et al *.* [45] explained about the thermal stability of the sulfones over the sulfinates. According to their studies, PhSO _2_ − reacts with highly stabilized benzhydrylium ions to give sulfone derivatives exclusively, but in the case of highly reactive benzhydrylium ions it gives mixtures of sulfinates Ar _2_ CH-OS(O)Ph and sulfones Ar _2_ CH-SO _2_ Ph; the latter rearranges to the thermodynamically more stable sulfones through an ionization recombination sequence.

**Scheme 2:**
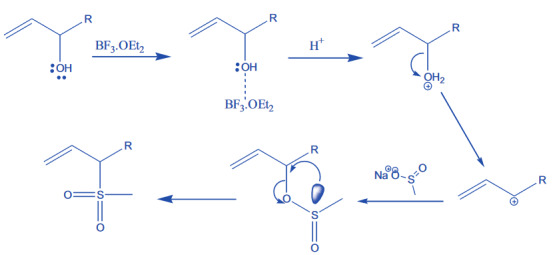
Possible reaction mechanism for conversion of alcohol into sulfones.

In the given Scheme 2, the reaction mechanism was explained schematically. Using acetic acid as a solvent instead of dichloromethane favors reaction at room temperature. Initially, BF _3_ .OEt _2_ activates the hydroxyl group to become protonated and subsequent elimination of water molecule occurs. As a result carbonium ion formation took place at room temperature itself. The formed carbonium ion was attacked by nucleophile of the sodium methyl sulfinates leading to the formation of sulfinate derivatives. Later, the product rearranged into thermodynamically more stable sulfones.

We chose the benzyl alcohol, and sodium methyl sulfinates as substrates for optimization of the reaction initially in the presence of BF _3_ .OEt _2_ .The authors carried out a couple of reactions by changing the concentration of BF _3_ .OEt _2_ , varying from 0.2 equivalents to 2.0 equivalents. Finally, we could achieve the yields of the target molecule variables from 15 to 92% as shown in Table 1.

**Table 1:** Reaction conditions for optimization.

Entry	BF _3_ .Et _2_ O	Solvent	T °C	Time (h)	Yield (%)
1	0.2	CH _2_ Cl _2_	50	3	40
2	1.0	CH _3_ COOH	28	3	80
3	1.4	CH _3_ COOH	28	3	82
4	1.6	CH _3_ COOH	28	3	86
5	1.8	CH _3_ COOH	28	3	92
6	2.0	CH _3_ COOH	28	3	90
7	1.8	DMSO	30	3	25
8	1.8	THF	28	3	20
9	1.8	CHCl _3_	28	3	37
10	1.8	Cyclohexane	28	3	15
11	1.8	CH _3_ NO _2_	28	3	39
12	1.8	C _2_ H _5_ NO _2_	28	3	40
13	1.8	1,4-Dioxane	28	3	42
14	1.8	CH _3_ CN	28	3	25
15	1.8	DMF	28	3	38
16	1.8	Acetone	28	3	30

The highest yields of the compound were obtained with 1.8 equivalents of BF _3_ .OEt _2_ . A couple of reactions were conducted with 1.8 equivalents of BF _3_ .OEt _2_ at various time periods ranging from 1 to 8 h. During the time period from 1 to 3 h, the yields found increased, and when the time period was prolonged from 3 to 8 h, the yields decreased. This reaction conversion was tremendously effective on the solvent which was used. For this reason, several trial reactions were carried out with both the polar solvents and nonpolar solvents. Lesser yields were reported with the nonpolar solvent cyclohexane (Table 1, entry 10). Next to cyclohexane, polar solvents like DMSO, and THF gave the yields of 25%, and 20% respectively. With the exception of acetic acid other solvents got the yields of the desired product below 50%. The best yields ranging from 80% to 92% (Table 1, entries 2–6) were obtained with the acetic acid. So, finally, we have concluded that the reaction is more favorable with the protic solvents.

The above optimized reaction conditions were verified and or generalized with structurally different types of alcohols. The obtained yields of desired products were mentioned in Table 2, entries 1–9.

The best yield (95%) of the desired molecule was obtained with the nitro alcohol derivatives (3i), under the optimized reaction condition. The lowest yields were obtained with the electron donating groups, which existed in the substrates. Electron withdrawing groups, which were present in the substrate molecules favor the conversion with excellent yields. The alcohol 1a having three donation groups present in the ortho and para position gave less yield (Table 2, entry 1). Due to the presence of orthosteric effect, alcohol derivatives 3a and 3e gave less yields (Table 2, entries 1, 5). The fewer number of donating groups present in the alcohol substrates increase the yields from 85% to 88%. The phenyl ring has a lesser withdrawing effect than the nitro group results and yields almost the highest yields.

**Table 2:** Sulfonation of various alcohols with sodium methyl sulfinates.

Entry	Compound structure	Number	Yield (%) ^a^
1	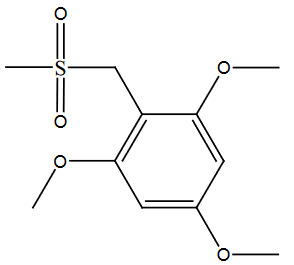	3a	85
2	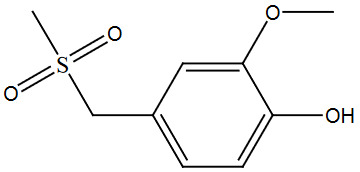	3b	88
3	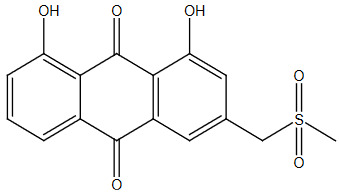	3c	90
4	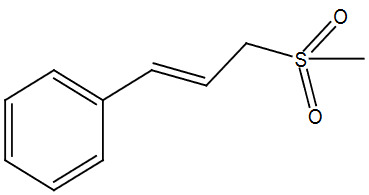	3d	91
5	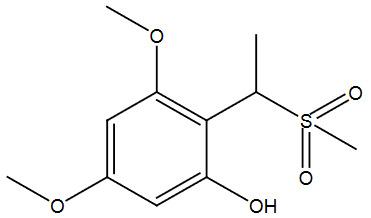	3e	88
6	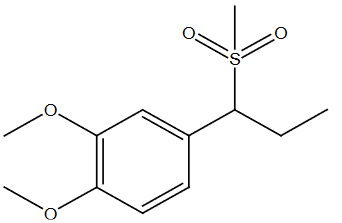	3f	86
7	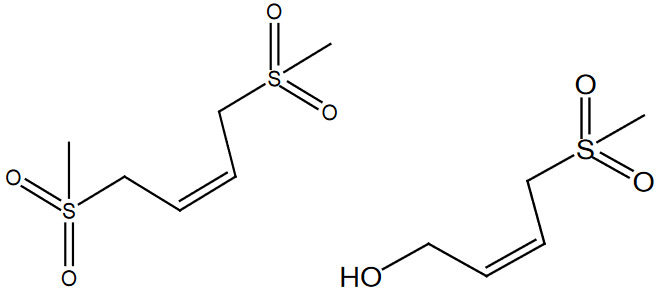	3g	88
8	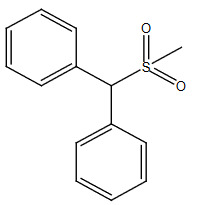	3h	92
9	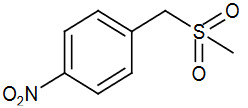	3i	95

aYields refer to pure products after column chromatography Reaction conditions: 1a–1i (1.96 μmol), 2 (1.96 μmol), BF _3_ .OEt _2_ (3.5 mL), Acetic acid (3 mL), at rt for 3h.

## 3. Biological activity

All the synthesized compounds were screened for antimicrobial activity and results were depicted in Table 3. Among the screened compounds (3a–3i), compound 3f with dimethoxy, hydroxyl benzyl group showed the highest inhibition zone followed by compounds 3a, 3c, and 3h. Further, the compound 3i was found to be effective on fungal strains. The remaining compounds showed moderate activity.

**Table 3:** Antimicrobial activity of the synthesized compounds (3a–3i).

	Diameter of zone of inhibition in mm																	
Compound Code	S.aureus (ATCC 25923)			B. Cereus (ATCC			P. Aeruginosa (ATCC 27853)			E.coli (ATCC 35218)			C. Albicans (ATCC 90028)			A. Niger (NCCS 1196)		
	50 (mg/mL)	100 (mg/mL)	150 (mg/mL)	50 (mg/mL)	100 (mg/mL)	150 (mg/mL)	50 (mg/mL)	100 (mg/mL)	150 (mg/mL)	50 (mg/mL)	100 (mg/mL)	150 (mg/mL)	50 (mg/mL)	100 (mg/mL)	150 (mg/mL)	50 (mg/mL)	100 (mg/mL)	150 (mg/mL)
3a	-	11	18	-	-	-	08	17	23	08	12	18	08	12	16	14	16	14
3b	08	12	17	-	-	-	07	15	21	09	13	17	08	13	14	13	15	13
3c	07	12	18	-	-	-	08	16	23	10	12	18	09	13	16	13	15	13
3d	06	13	17	-	-	-	07	13	16	08	12	15	09	14	17	14	15	14
3e	07	14	18	-	-	-	08	16	20	09	13	18	10	14	16	14	16	16
3f	08	16	19	-	-	-	10	17	24	09	14	18	09	16	17	15	17	16
3g	07	14	16	-	-	-	08	12	15	08	14	17	07	14	15	15	15	15
3h	07	13	18	-	-	-	07	18	23	08	15	18	08	16	16	14	15	15
3i	06	14	17	-	-	-	06	12	16	08	14	17	11	14	18	15	17	18
Ciproflaxacin(30 mg/disc)	24			18			24			23			NA			NA		
Fluconazole(25 µg/disc)	NA			NA			NA			NA			22			20		

## 4. Conclusion

This method is a modified method for methyl sulfones and reaction yields of 85% to 95% were obtained. In this method, the solvent acetic acid was used, which is inexpensive when compared with the solvent dichloromethane solvent, and this reaction is carried out at room temperature. We have applied this method for the synthesis of 9 compounds of which 7 are novel (3a–3i).

The compounds bearing dimethoxy, hydroxyl benzyl group have shown prominent antibacterial activity when compared to compounds without these groups. It was also confirmed that the compounds bearing nitro group have shown prominent antifungal activity when compared to other compounds. Further investigation in this area may help to create more potent drugs for the treatment of bacterial and fungal infections.

## 5. Experimental section

### 5.1. General preparation of compounds (1a–1i)

NaBH _4_ (4.76 μmol) was added to the ethyl alcohol (3 mL) and the reaction mixture was stirred at room temperature for 5 min. Respectively, aldehyde compound (4.76 μmol) was added to the reaction mixture and stirred continuously for 1 h. Reaction mixture completion was confirmed by the TLC. After completion of the reaction, the mixture was quenched with 10% HCl (3 mL) and ethanol was evaporated under reduced pressure. After the complete removal of ethanol, saturated sodium bisulfite (1 × 5 mL) was added. The organic compound was extracted with dichloromethane (20 mL) and water (10 mL). The organic layer was dried over Na _2_ SO _4_ , filtered, and concentrated under reduced pressure; to give 1a–1i compounds. Yield, ^1^ H NMR, ESI-MS (M+H) data of all compounds, and CHNS/O u1d57 (Perkin-Elmer 2400, PerkinElmer Inc., Waltham, MA, USA) composition data of each product are given below.

### 5.1.1. (2,4,6-trimethoxyphenyl)methanol (1a)

Brown solid, yield 91.2%; ^1^ H NMR (CDCl _3_ , 400 MHz): *δ* 6.12 (s, 2H), 4.69 (s, 2H), 3.81 (s, 6H), 3.80 (s, 3H), 2.16 (s, ^1^ H,); ESI MS (M+H): *m/z* 199.01.

### 5.1.2. 4-(hydroxymethyl)-2-methoxyphenol (1b)

White solid, ^1^ H NMR (CD _3_ OD, 400 MHz): *δ* 6.95 (s, ^1^ H), 6.79 (s, 2H), 4.52 (s, 2H), 3.85 (s, 3H); ^13^ C NMR (CD _3_ OD, 100 MHz): *δ* 147.54, 145.47, 132.86, 119.75, 114.66, 110.79, 63.98, 55.04; ESI MS (M+H): *m/z* 155.26.

### 5.1.3. 1,8-dihydroxy-3-(hydroxymethyl)anthracene-9,10-dione (1c)

Pale white solid, ^1^ H NMR (DMSO-d6, 100 MHz): *δ* 11.90 (s, 2H), 7.79–7.64 (m, 3H), 7.35–7.24 (m, 2H), 5.57 (t, *J* = 5.8 Hz, ^1^ H), 4.57 (d, *J* = 5.8 Hz, 2H); ESI MS (M+H): *m/z* 135.19.

### 5.1.4. 3-phenylprop-2-en-1-ol (1d)

Light yellow solid, ^1^ H NMR (CDCl _3_ , 500 MHz): *δ* 7.36-7.32 (m, 2H), 7.27–7.24 (m, 2H), 7.23–7.19 (m, ^1^ H), 6.59 (s, ^1^ H), 6.55 (s, ^1^ H), 4.44–4.42 (m, 2H); ESI MS (M+H): *m/z* 135.19.

### 5.1.5. 2-(1-hydroxyethyl)-3,5-dimethoxyphenol (1e)

Yellow solid, ^1^ H NMR (CDCl _3_ , 400 MHz): *δ* 5.85 (s, ^1^ H), 5.82 (s, ^1^ H), 4.61 (s, ^1^ H), 4.0 (s, ^1^ H), 3.82 (s, ^1^ H), 3.65 (s, 6H), 1.55 (s, 3H); ESI MS (M+H): *m/z* 199.06.

### 5.1.6. 1-(3,4-Dimethoxyphenyl)-1-propanol (1f)

Brown solid, ^1^ H NMR (CDCl _3_ , 400 MHz): *δ* 7.05 (d, ^1^ H), 6.94–6.85 (dd, *J* = 8.1 Hz, 2H), 4.85 (m, ^1^ H), 3.88 (ds, 6H), 2.45 (bs, OH), 1.84–1.78 (m, 2H), 0.97 (t, *J* = 7.4 Hz, 3H); ESI MS (M+H): *m/z* 197.29.

### 5.1.7. But-2-ene-1,4-diol (1g)

White solid, ^1^ H NMR (CDCl _3_ , 400 MHz): *δ* 5.80 – 5.78 (m, 2H), 4.25–4.24 (m, 4H); ESI MS (M+H): *m/z* 89.29.

### 5.1.8. Diphenylmethanol (1h)

White solid, ^1^ H NMR (CDCl _3_ , 500 MHz): *δ* 2.37 (bs, ^1^ H), 5.79 (s, ^1^ H), 7.25–7.36 (m, 10H); ^13^ C NMR (CDCl _3_ , 125 MHz): *δ* 143.7, 128.4, 127.5, 126.5, 76.2; ESI MS (M+H): *m/z* 185.01.

### 5.1.9. (4-nitrophenyl) methanol (1i)

Light yellow solid, ^1^ H NMR (CDCl _3_ , 300 MHz): *δ* 8.23 (d, 2H, *J* = 8.7), 7.54 (d, 2H, *J* = 8.7), 4.84 (s, 2H); ^13^ C NMR (CDCl _3_ , 75.47 MHz): *δ* 148.08, 126.99, 123.73, 64.02; ESI MS (M+H): *m/z* 154.15.

### 5.2. General preparation of compounds (3a-i)

The respective benzyl alcohol (1.96 μmol) was dissolved in acetic acid (3 mL). BF _3_ .OEt _2_ (3.5 mL, 3.528 μmol) was added to the reaction mixture at room temperature. Sodium methyl sulfinate (200 mg, 1.96 μmol) was added to the reaction mixture and stirred for 30 min. Reaction mixture completion was confirmed by the TLC. After completion of the reaction, the reaction mixture was quenched with NaHCO _3_ solution (10 mL). The organic compound was extracted with dichloromethane (20 mL) and water (10 mL). The organic layer was dried over Na _2_ SO _4_ , filtered, and concentrated under reduced pressure. The crude material was purified by the silica gel chromatography to give the compounds 3a–3i. Yield, IR, NMR, ESI MS (M+H) data, and CHNS/O u1d5a (Perkin-Elmer 2400) data of each product are given below.

### 5.2.1. 1,3,5-trimethoxy-2-((methylsulfonyl)methyl)benzene (3a)

Brown solid, yield 85%; IR (u, KBr): 3033 (Aromatic C=C), 2988, 2976 (CH _3_ ),1024(SO _2_ -) cm ^-1^ ; ^1^ H NMR (400 MHz, CDCl _3_ ): *δ* 6.16 (s, 2H), 4.38 (s, 2H), 3.85 (s, 6H), 3.82 (s, 3H). 2.77 (s, 3H); ^13^ C NMR (100 MHz, CDCl _3_ ): 162.23, 159.72, 98.86, 91.03, 90.44, 56.04, 55.94, 55.45, 50.26, 40.31; ESI MS: *m/z* 181 (M-SO _2_ Me).CHNS: Anal. calcd. for C _11_ H _16_ O _5_ S; C, 50.75; H, 6.20; S, 12.32. Found: C, 50.61; H, 6.11; S, 12.49.

### 5.2.2. 2-methoxy-4-((methylsulfonyl)methyl)phenol (3b)

Brown solid, yield 88%; IR (u,KBr): 3329 (phenolic OH),2888, 2785 (CH _3_ ), 1020 (SO _2_ -) cm ^-1^ ; ^1^ H NMR (400 MHz, CDCl _3_ ) *δ* 6.70–6.67 (m, 3H), 4.41 (s, 2H), 3.83 (s, 3H), 3.46 (s, ^1^ H), 2.96 (s, 3H); ^13^ C NMR 147.84, 147.64, 124.83, 118.45, 117.39, 117.32, 58.97, 56.79, 41.75; ESI MS: *m/z* 202 (M+-SO _2_ ).CHNS: Anal. calcd. for C _9_ H _12_ O _4_ S; C, 49.99; H, 5.59; S, 14.83. Found: C, 49.88; H, 5.43; S, 14.96.

### 5.2.3. 1,8-dihydroxy-3-((methylsulfonyl)methyl)anthracene-9,10-dione (3c)

Brown solid, yield 90%; IR (u,KBr): 3321 (phenolic OH), 3040 (Aromatic C=C), 1020 (SO _2_ -) cm ^-1 ^; ^1^ H NMR (400 MHz, CDCl _3_ ): *δ* 12.06 (s, ^1^ H), 12.04 (s, ^1^ H), 7.85- 7.83 (m, ^1^ H), 7.78 (s, ^1^ H), 7.71-7.67 (t , *J* = 16 Hz, ^1^ H), 7.32-7.30 (d, ^1^ H), 7.26 (s, ^1^ H), 5.19 (s, 2H), 2.19 (s, 3H); ^13^ C NMR (100 MHz, CDCl _3_ ): *δ* 192.69, 181.48, 170.38, 162.82, 146.52, 137.29, 133.93, 133.60, 124.76, 122.39, 120.15, 118.49, 115.85, 115.32, 64.70, 20.77; ESI MS (M++2H): *m/z* 333.CHNS: Anal. calcd. for C _16_ H _12_ O _6_ S; C, 57.83; H, 3.64; S, 9.65. Found: C, 57.91; H, 3.49; S, 9.81.

### 5.2.4. 1-((E)-3-(methylsulfonyl)prop-1-enyl)benzene (3d)

Brown solid, yield 91%; IR (u,KBr): 3033 (Aromatic C=C), 2970, 2965 (CH _3_ ), 1024 (SO _2_ -) cm ^-1 ^; ^1^ H NMR (400 MHz, CDCl _3_ ): *δ* 7.31–7.29 (m, 2H), 7.25–7.22 (m, 2H), 7.19– 7.17 (m, ^1^ H), 6.56–6.53 (m, ^1^ H), 6.22–6.16 (m, ^1^ H), 4.09–4.07 (d, *J* = 8, 2H), 3.00 (s, 3H); ^13^ C NMR (100 MHz, CDCl _3_ ): *δ* 137.67, 136.33, 129.19, 128.08, 127.08, 122.13, 58.35, 40.25; ESI MS: *m/z* 118 (M+-SO _2_ ).CHNS: Anal. calcd. for C _10_ H _12_ O _2_ S; C, 61.20; H, 6.16; S, 16.34. Found: C, 61.09; H, 6.03; S, 16.47.

### 5.2.5. 3,5-dimethoxy-2-(1-(methylsulfonyl)ethyl)phenol (3e)

Brown solid, yield 86%; IR (u,KBr): 3041 (Aromatic C=C), 2980, 2889 (CH _3_ ), 1024 (SO _2_ -) cm ^-1 ^; ^1^ H NMR (400 MHz, CDCl _3_ ): *δ* 6.20–6.15 (m, 2H), 4.40–4.36 (m, ^1^ H), 3.82 (s, 3H), 3.80 (s, 3H), 2.97 (s, 3H), 1.79 (s, 3H); ^13^ C NMR (100 MHz, CDCl _3_ ): *δ* 167.49, 164.44, 162.99, 113.34, 93.61, 91.47, 62.29, 56.79, 56.04, 37.80, 17.52; ESI MS (M+H): *m/z* 258(M+).CHNS: Anal. calcd. for C _11_ H _16_ O _5_ S; C, 50.75; H, 6.20; S, 12.32. Found: C, 50.60; H, 6.11; S, 12.47.

### 5.2.6. 1,2-dimethoxy-4-(1-(methylsulfonyl)propyl)benzene (3f)

Brown solid, yield 88%; IR (u,KBr): 3033 (Aromatic C=C), 2988, 2972, 1024 (SO _2_ -) cm ^-1 ^; ^1^ H NMR (400 MHz, CDCl _3_ ) *δ* 6.91 (s, ^1^ H), 6.84–6.82 (m, 2H), 4.13–4.11 (m, ^1^ H), 3.82 (s, 6H), 3.03(s, 3H), 2.25–2.20 (m, 2H), 1.04-1.01(s, 3H); ^13^ C NMR (100 MHz, CDCl _3_ ): *δ* 149.53, 147.02, 135.91, 117.05, 116.41, 113.45, 69.90, 56.78,39.66, 23.95, 11.02; ESI MS (M+H): *m/z* 215(M+).CHNS: Anal. calcd. for C _12_ H _18_ O _4_ S; C, 55.79; H, 7.02; S, 12.42. Found: C, 55.65; H, 6.89; S, 12.54.

### 5.2.7. (Z)-1,4-bis(methylsulfonyl)but-2-ene, (Z)-4-(methylsulfonyl)but-2-en-1-ol (3g)

Brown solid, yield 88%; IR (u,KBr): 3323 (-OH), 1020 (SO _2_ -) cm ^-1 ^; ^1^ H NMR (400 MHz, CDCl _3_ ): *δ* 5.83–5.82 (m, ^1^ H), 5.72–5.70 (m, 3H), 4.67–4.63 (m, 4H), 4.55–4.54 (m, 2H), 2.04 (s, 3H), 2.03 (s, 6H); ESI-MS (M+H): *m/z* 261 (M+).

### 5.2.8. (methylsulfonyl)diphenylmethane(3h) [31]

Brown solid, yield 92%; ^1^ H NMR (400 MHz, CDCl _3_ ): *δ* 7.65–7.57 (m, 4H), 7.43–7.33 (m, 6H), 5.32 (s, ^1^ H), 2.77 (s, 3H); ^13^ C NMR (100 MHz, CDCl _3_ ): *δ* 132.80, 129.73, 129.66, 128.50, 74.84, 40.02; ESI MS (M+H): *m/z* 247. CHNS: Anal. calcd. for C _14_ H _14_ O _2_ S; C, 68.26; H, 5.73; S, 13.02. Found: C, 68.17; H, 5.61; S, 13.16.

### 5.2.9. 1-((methylsulfonyl)methyl)-4-nitrobenzene (3i) [46]

Brown solid, yield 95%; ^1^ H NMR (400 MHz, CDCl _3_ ) δ8.44 (d, J= 8.8 Hz, 2H), 8.16(d, J= 8.8 Hz, 2H), 3.12 (s, 3H); ^13^ C NMR (100 MHz, CDCl _3_ ): δ 150.9, 145.86, 129.25, 124.36, 44.29; ESI MS (M+H): m/z198.CHNS: Anal. calcd. for C _8_ H _9_ NO _4_ S; C, 44.64; H, 4.21; N, 6.51; S, 14.90. Found: C, 44.51; H, 4.12; N, 6.51; S, 14.99.

### 5.3. Antibacterial activity [47]

The antibacterial activity of the compounds was determined by means of the disc diffusion method. Cultures of each bacterium ( *E.coli, Bacillus cereus, Staphylococcus aureus, and Pseudomonas aeruginosa)* were inoculated to the nutrient broth and incubated at 37 °C for 16 h., respective bacterial culture was inoculated in the MHA plate by using the spread plate method. Discs (6 mm in diameter) were impregnated with 25, 50, and 75 µg/ mL concentrations in DMSO solution of the compounds **(3a–3i)** and placed on the surface of the MHA inoculated with bacteria, which were incubated at 37 °C for 24 h. The inhibition zones were measured with a caliper considering the total diameters. Similarly, each plate carried a blank disc, the disk with DMSO, and ciprofloxacin disc (30 µg/mL) as standard.

## 5.4. Antifungal activity

The antifungal activity of the compounds was determined by means of the disc diffusion method. Cultures of each fungal ( *C.Albicans, and A. niger)* were inoculated to the nutrient broth and incubated at 37 °C for 16 h. Respective fungal culture was inoculated in the SDA plate by using the spread plate method. Discs (6 mm in diameter) were impregnated with 25, 50, and 75 µg/ mL concentrations in DMSO solution of the compounds **(3a–3i)** and placed on the surface of the MHA inoculated with bacteria, which were incubated at 37 °C for 24 h. The inhibition zones were measured with a caliper considering the total diameters. Similarly, each plate carried a blank disc, disc with DMSO, and fluconazole disc (30 µg/mL) as standard.
